# N-methyl-D-aspartate receptor availability in first-episode psychosis: a PET-MR brain imaging study

**DOI:** 10.1038/s41398-021-01540-2

**Published:** 2021-08-12

**Authors:** Katherine Beck, Atheeshaan Arumuham, Mattia Veronese, Barbara Santangelo, Colm J. McGinnity, Joel Dunn, Robert A. McCutcheon, Stephen J. Kaar, Nisha Singh, Toby Pillinger, Faith Borgan, James Stone, Sameer Jauhar, Teresa Sementa, Federico Turkheimer, Alexander Hammers, Oliver D. Howes

**Affiliations:** 1 Department of Psychosis Studies, Institute of Psychiatry, Psychology & Neuroscience, Kings College London, De Crespigny Park, London, SE5 8AF UK; 2 Psychiatric Imaging Group, MRC London Institute of Medical Sciences, Hammersmith Hospital, London, W12 0NN UK; 3 South London and Maudsley NHS Foundation Trust, London, UK; 4 Institute of Clinical Sciences (ICS), Faculty of Medicine, Imperial College London, London, W12 0NN UK; 5 Centre for Neuroimaging Sciences, Institute of Psychiatry, Psychology and Neuroscience, King’s College London, London, UK; 6 King’s College London & Guy’s and St Thomas’ PET Centre, School of Biomedical Engineering & Imaging Sciences, King’s College London, St Thomas’ Hospital, London, SE1 7EH UK; 7 Brighton and Sussex Medical School, University of Sussex, Falmer, Brighton, UK; 8 Present Address: COMPASS Pathways plc, London, UK

**Keywords:** Schizophrenia, Molecular neuroscience

## Abstract

N-methyl-D-aspartate receptor (NMDAR) hypofunction is hypothesised to underlie psychosis but this has not been tested early in illness. To address this, we studied 40 volunteers (21 patients with first-episode psychosis and 19 matched healthy controls) using PET imaging with an NMDAR selective ligand, [^18^F]GE-179, that binds to the ketamine binding site to index its distribution volume ratio (DVR) and volume of distribution (*V*_T_). Hippocampal DVR, but not *V*_T_, was significantly lower in patients relative to controls (*p* = 0.02, Cohen’s *d* = 0.81; *p* = 0.15, Cohen’s *d* = 0.49), and negatively associated with total (rho = −0.47, *p* = 0.04), depressive (rho = −0.67, *p* = 0.002), and general symptom severity (rho = −0.74, *p* < 0.001). Exploratory analyses found no significant differences in other brain regions (anterior cingulate cortex, thalamus, striatum and temporal cortex). These findings are consistent with the NMDAR hypofunction hypothesis and identify the hippocampus as a key locus for relative NMDAR hypofunction, although further studies should test specificity and causality.

## Introduction

Schizophrenia is a chronic mental illness with a lifetime prevalence of 1% [1]. It has been ranked the third most disabling illness worldwide [2]. Several lines of evidence indicate that the pathophysiology of schizophrenia and related psychotic disorders involves N-methyl-D-aspartate receptor (NMDAR) hypofunction [3–7]. This hypothesis was initially proposed in the 1990s [8, 9] on the basis of observations that ketamine and phencyclidine (PCP) induced the full range of schizophrenia-like symptoms (positive, negative and cognitive) when given to healthy participants; and evidence that these compounds worsen symptoms in patients with schizophrenia [10–12]. Ketamine and PCP show uncompetitive NMDAR antagonism through binding to an intrachannel binding site to block the active channel [13].

Genome-wide association studies have identified genetic variants related to the structure and function of the NMDAR that are associated with schizophrenia, including GRIN2A and serine racemase (SRR) [14]. Mice with reduced NMDAR expression display schizophrenia-like phenotypes [15–17]. There is also evidence from post-mortem studies that the NMDAR density is reduced in the hippocampus of humans with schizophrenia [18]. While these studies indicate the NMDAR is altered in schizophrenia, the only in vivo imaging study of the NMDAR in schizophrenia to date used [^123^I]CNS-1261, a single-photon emission tomography (SPECT) radioligand, selective for the intrachannel ketamine/PCP binding site [19, 20]. This study found a lower ratio of tracer binding in the left hippocampus to that in the whole brain in antipsychotic-free patients relative to healthy participants. Moreover, the hippocampal binding ratio was negatively correlated with total and negative symptom severity in antipsychotic-treated patients. However, as the study was conducted in chronic patients, it is unknown if NMDAR alterations are present early in the illness or if they may be secondary to the effects of illness chronicity.

In view of this, we investigated the in vivo availability of NMDAR in patients with first-episode psychosis (FEP). We hypothesized that patients would show lower NMDAR availability in the hippocampus relative to matched healthy controls; and that NMDAR availability would be negatively correlated with symptom severity. In addition, as NMDAR availability has been found to be reduced in patients with epilepsy receiving antidepressants for depressive symptoms [21], we tested whether there was an association between NMDAR availability and depressive symptom severity.

## Methods

### Ethics statement

Approval was obtained by the West London & GTAC Research Ethics Committee (REC reference: 16/LO/0130) and the Administration of Radioactive Substances Advisory Committee. Volunteers demonstrated capacity and provided written informed consent to participate. We followed the Strengthening the Reporting of Observational Studies in Epidemiology (STROBE) reporting guidelines for case-control studies.

### Participants

Data were collected from November 7, 2016 until August 2, 2019. Patients were recruited from FEP services in London, United Kingdom. Inclusion criteria were as follows: a diagnosis of a *DSM-IV* psychotic disorder according to the *Structured Clinical Interview of DSM-IV-TR Axis I Disorders-Patient Edition* [22], fulfilling criteria for having the first episode of psychosis [23], and less than 5 years’ illness duration. For comparison, a sample of healthy controls matched on age (+/−3 years) and sex were included. Inclusion criteria were as follows: no current or lifetime history of Axis I Disorder as determined by the *Structural Clinical Interview of DSM-IV-TR Axis I Disorders-Patient Edition* [22].

Exclusion criteria for all volunteers were as follows: history of significant head trauma (such as loss of consciousness >1 min or requiring hospital admission), dependence on illicit substances or alcohol, positive urine drug test (SureScreen Diagnostics, Derby, UK) for any illicit substances that might affect NMDAR (e.g. stimulants) on the day of scanning, medical comorbidity (other than minor illnesses), current use (no use within 3 months) of any of the following drugs which may interfere with NMDAR; antidepressants [21], mood stabilizers [24–26], benzodiazapines [27]; and contraindications to scanning (such as pregnancy) (see eMethods 1 in the Supplement for full exclusion criteria).

#### Medication status

Participants with psychosis were classified as antipsychotic-free if they had been free from antipsychotic treatment for at least 6 weeks for oral or 6 months for depot formulations [28]. Antipsychotic-naive was defined as having had no antipsychotic treatment at all.

In total *n* = 32 patients were screened for eligibility, *n* = 25 were deemed eligible, and *n* = 21 were included in the study. *N* = 36 healthy volunteers were screened for eligibility as controls, *n* = 34 were deemed eligible, and *n* = 19 were included in the study. Participants who were deemed eligible, but were not included in the study, withdrew following screening prior to scanning.

The final sample included *n* = 40 individuals, including *n* = 19 healthy controls and *n* = 21 patients (*n* = 12 antipsychotic-free and *n* = 9 treated with antipsychotics).

### Measures

#### Clinical and demographic variables

Current age and illness duration were recorded. Clinical symptom severity was determined using the Positive and Negative Syndrome Scale (PANSS) [29]. Psychotropic medication histories were recorded, urine drug screens were performed, and equivalent chlorpromazine doses were calculated using the method reported by Leucht et al. [30].

### Neuroimaging

All participants underwent a dynamic, continuous 90-min simultaneous PET-MR acquisition after a bolus injection of [^18^F]GE-179 (mean [SD], 140.31 [9.04] MBq) with a Siemens 3T Biograph mMR PET/MR hybrid scanner (Siemens, Erlangen, Germany). In parallel to PET/MR imaging, continuous arterial sampling using an MR compatible blood sampler (http://www.swisstrace.ch/blood-sampler-twilite.html) was performed for the first 16 min followed by 6 discrete samples (see eMethods 2 in the Supplement for the full acquisition protocol). A T1-weighted structural Magnetization Prepared Rapid Gradient-Echo (MP-RAGE) image was acquired for co-registration (see eMethods 3 in the Supplement for the sequences). At the end of the session, a separate low dose CT scan (140 kV, 10 mA, helical acquisition) of the subject’s head was acquired on a GE Discovery DST 710 PET/CT (GE Healthcare, Chicago, Illinois, USA), and used for tissue attenuation correction during the PET image reconstruction. This additional CT scan was required because, at the time of the study starting, MRI based methods for attenuation correction had not been validated.

The person conducting the image analysis was blinded to the group status of the volunteers. NMDAR availability was determined as the [^18^F]GE-179 volume of distribution (*V*_T_, mL/cm^3^) calculated using the standard 2-tissue compartmental modelling method with a metabolite-corrected arterial plasma input function, consistent with previous [^18^F]GE-179 brain PET studies [31] (see eMethods 4–9 in the Supplement for more information on PET image analysis and model validation). Prior to kinetic modelling, all the individual PET data underwent the same image processing pipeline to measure and correct for subject motion, segment brain tissues and extract [^18^F]GE-179 tracer activity in the main regions of interest. For comparison with previous results [19], PET analysis was performed using the distribution volume ratio (DVR), calculated for the aforementioned regions as follows: region of interest *V*_T_/*V*_T_. The hippocampus was the primary region of interest given the prior findings of Pilowsky et al. [19] and evidence implicating it in the pathophysiology of psychotic and cognitive symptoms [18, 32, 33]. Exploratory analyses were conducted in the following additional ROIs: the anterior cingulate cortex (ACC), thalamus, striatum, and temporal lobe. These regions were chosen because prior studies have shown that patients with schizophrenia show glutamatergic dysfunction in these regions [18, 34]. Further exploratory analyses were completed to compare hippocampal DVR and *V*_T_ across subgroups (antipsychotic-free patients, antipsychotic-treated patients and healthy controls). To determine if volumetric group differences influenced our findings, hippocampal volumes were compared across groups (eMethod 7).

Exploratory voxel-wise analyses were also conducted to determine if there were alterations in brain regions outside of the regions of interest using [^18^F]GE-179 *V*_T_ parametric maps derived using the Logan graphical approach [35]. The main outputs from the image analysis were manually quality controlled (QC) by an experienced PET modeller (MV), blinded to subject status (for full description see eMethods 4 PET image analysis in the Supplement). Participants were excluded on the basis that there were still obvious artefacts on the motion corrected images and TACs (e.g. frame misalignment and signal drops in particular frames (for full description see eMethods 4 PET image analysis in the Supplement). Scans which failed QC were not included in the statistical analysis.

### Statistical analysis

Statistical Product and Service Solutions (SPSS) version 22 (IBM Corp) was used for all statistical analyses and the significance level was set to *p* < 0.05 (two-tailed). Data normality was assessed using the Shapiro–Wilk test. Categorical clinical, demographics, and experimental variables were compared across groups using *χ*^2^ tests; and continuous variables were assessed using independent samples *t*-tests and Mann–Whitney tests for parametric and non-parametric data, respectively.

To determine whether hippocampal tracer uptake was lower in patients as compared to controls, an independent samples *t*-test was used. Mann–Whitney tests were used to analyze DVR data as they were non-normally distributed. To explore if there were effects in other regions, independent *t*-tests and Mann–Whitney tests were again used to identify if other areas known to be involved in schizophrenia had different tracer uptake. Independent *t*-tests and Mann–Whitney tests were also used for exploratory analyses to compare hippocampal DVR and *V*_T_ across subgroups (antipsychotic-free patients, antipsychotic-treated patients and healthy controls) and to compare hippocampal volumes between the patient and healthy control groups. None of the exploratory analyses (additional ROIs or patient subgroup analyses) were corrected for multiple comparisons as they were exploratory. Effect sizes were summarised as Cohen’s *d*, and calculated using the difference in means between groups and the pooled standard deviation of the patient and control groups. The coefficient of variation was calculated for the *V*_T_ and DVR of the hippocampus and other exploratory regions, in both patient and control groups, by calculating the ratio of the standard deviation to the mean (see eTable 1).

**Table 1:** Clinical and demographic variables.

Characteristic	Healthy volunteers (*n* = 18)	Patients with FEP (*n* = 19)
Age, years, mean (SD)	26.7 (4.5)	25.3 (4.9)
Sex, No. male/female	14/4	15/4
Diagnosis, schizophrenia (*n*)/schizoaffective disorder (*n*)		19/0
Illness duration, months, mean (SD)		23 (16.5)
Antipsychotic-free/naive (*n*)		8/3
For patients taking antipsychotic treatment; chlorpromazine equivalent dose (mg/d), mean (SD)		393 (166)
Current antipsychotic medication (*n*): Aripiprazole:Apripirazole&Olanzapine:Risperidone:Lurasidone		4:1:2:1
PANSS positive score, mean (SD)^a^		19.2 (4.4)
PANSS negative score, mean (SD)^a^		19.6 (5.7)
PANSS general score, mean (SD)^a^		36.6 (7.3)
PANSS total score, mean (SD)^a^		75.4 (11.1)
PANSS depression item of rating, mean (SD)		2.4 (1.5)

*FEP* first-episode psychosis, *Shaded box* not applicable, *PANSS* Positive and Negative Syndrome Scale, *SD* standard deviation, *n* number.

^a^Scores range for total PANSS: 53–88, general PANSS subscale: 25–46, positive subscale of the PANSS: 12–27; negative subscale of the PANSS: 8–28; and depression item: 1–6. Higher scores indicate greater symptom severity.

To investigate our hypothesis that tracer uptake was negatively associated with symptom severity, Pearson’s or Spearman’s correlation coefficients were calculated for normally and non-normally distributed data respectively, including PANSS total symptom severity as the independent variable, and tracer DVR or *V*_T_ as the dependent variables. We also performed secondary analyses to explore if there were relationships with the general, positive, and negative subscales, using Bonferroni corrections to adjust for multiple comparisons in these exploratory analyses. We used the same approach to test our exploratory hypothesis that NMDAR availability would be negatively associated with depressive symptoms, using the score on the depressive item of the PANSS. Spearman’s correlation coefficients were calculated to determine if there were associations between hippocampal DVR and antipsychotic dose and hippocampal volume [36]. To further determine whether volume effects affected the results we performed a one-way ANOVA with hippocampal DVR as the dependent variable and group (patient or control) as the independent variables with hippocampal volume as the covariate.

## Results

### Demographics and experimental variables

A total of *n* = 40 individuals participated in the study, including *n* = 19 healthy controls and *n* = 21 patients (*n* = 12 antipsychotic-free and *n* = 9 treated with antipsychotics). One healthy control was excluded due to a malfunction of the arterial line, and two patients were excluded due to a positive urine test for cocaine (*n* = 1), or QC failure (*n* = 1). A total of *n* = 37 individuals (*n* = 18 healthy controls and *n* = 19 patients, of whom *n* = 11 were antipsychotic-free) were included in the final analysis.

No significant group differences were found for age (*t*_35_ = 0.93, *p* = 0.36) (Table 1), hippocampal, or whole-brain tissue volume (see eTable 1 and eResults 1 in the Supplement), cumulative movement, input function, injected mass, injected activity, or molar activity (see eTable 2 in the Supplement).

### Tracer uptake in hippocampus

**Fig. 1:**
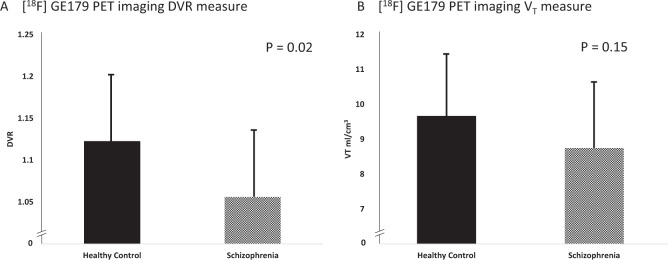
[^18^F]GE-179 DVR and *V*_T_ hippocampus. NMDAR availability measured by positron emission tomographic (PET) imaging was significantly lower in the hippocampus in patients with first-episode psychosis when DVR was measured (**A**) but not when *V*_T_ was measured (**B**). Data are expressed as mean (SD) of the distribution volume ratio (DVR) (**A**) and distribution volume (*V*_T_) (**B**) of [^18^F]GE-179.

The hippocampal DVR data were not normally distributed (*p* < 0.03). Hippocampal DVR was significantly lower in the patient sample (Mean Rank = 14.84) compared to healthy controls (Mean Rank = 23.39; Mann–Witney test: *U* = 92.00; *z* = −2.40, *p* = 0.02; Cohen’s *d* = 0.81; Fig. 1). The coefficients of variation in hippocampal DVR for the patient and control groups were 7.5% and 7.1%, respectively. We conducted sensitivity analyses to determine if hippocampal volume could influence our findings. These showed that there was no correlation between hippocampal volume and hippocampal DVR (rho −0.14, *p* = 0.4), and there was still a significant effect of group on hippocampal DVR when hippocampal volume was included as a covariate (F(1,34) = 5.8, *p* = 0.02).

When antipsychotic-free patients were compared with healthy controls, there was no significant difference in hippocampal DVR (Mann–Witney test: *U* = 68.00; *z* = −1.39 *p* = 0.17; eFigure 1 in Supplementary Material). However, hippocampal DVR was significantly lower in the patient group taking antipsychotics relative to healthy controls Mann–Witney test: *U* = 24.00; *z* = −2.67, *p* = 0.006; eFigure 1 in Supplementary Material). There was no significant difference between antipsychotic-free and treated patient groups (Mann–Witney test: *U* = 24.00; *z* = −1.65 *p* = 0.10; eFigure 1 in Supplementary Material). Furthermore, there was no significant correlation between antipsychotic dose and hippocampal DVR (rho = −0.16, *p* = 0.71).

Volume of distribution data were normally distributed (*p* = 0.63). While in absolute terms hippocampal *V*_T_ was lower in the patient group (Cohen’s *d* = 0.49), there was no statistically significant effect of group on *V*_T_ in the hippocampus (*t*_35_ = 1.49, *p* = 0.15; Fig. 1). The coefficients of variation in *V*_T_ for the patient and control groups were 22.7% and 18.2%, respectively. There were no significant differences in the comparisons between patients subgrouped on the basis of antipsychotic treatment and controls (antipsychotic-free vs healthy controls (*t*_27_ = −0.75, *p* = 0.46; eFigure 2 in Supplementary Material), antipsychotic-treated vs healthy controls (*t*_24_ = −1.94, *p* = 0.06; eFigure 2 in Supplementary Material), antipsychotic-free vs antipsychotic-treated (*t*_17_ = 0.94, *p* = 0.36; eFigure 2 in Supplementary Material).

### Tracer uptake in additional brain areas and whole brain

**Fig. 2:**
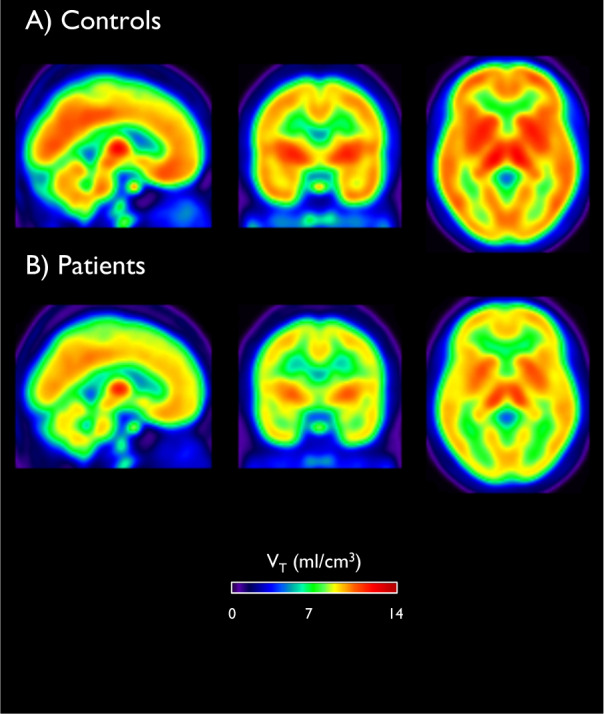
[^18^F]GE-179 *V*_T_. There was no significant difference in NMDAR availability in the hippocampus between patients and controls *t*_35_ = 1.49, *p* = 0.15 as determined by the distribution volume (*V*_T_; measured in millilitres per cubic centimetre). Images are mean parametric maps for controls (**A**), sample (**B**).

Figure 2 shows mean parametric maps of *V*_T_ of [^18^F]GE-179 for controls and patients, showing widespread uptake of the tracer throughout grey matter, in line with the expected distribution of NMDAR [31]. There was no significant effect of group on *V*_T_ or DVR of [^18^F]GE-179 when investigated in the exploratory ROIs (the ACC, thalamus, striatum or temporal lobe; see eTable 1 and eResults 2 in the Supplement). Similarly, the voxel-wise analysis did not identify significant group differences in the *V*_T_ of [^18^F]GE-179 in the hippocampus or other brain regions between patients and controls (see eResults 3 in the Supplement). There was no significant effect of group on *V*_T_ in the whole brain (*t*_35_ = 0.60, *p* = 0.56, Cohen’s *d* = 0.20).

### NMDAR availability and symptoms

**Fig. 3:**
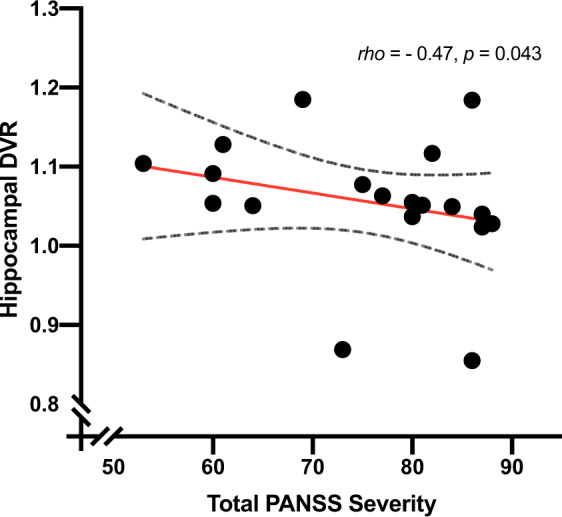
Relationship of tracer uptake in the hippocampus, as measured by DVR, and total PANSS symptom severity. Dashed line represents 95% CI.

**Fig. 4:**
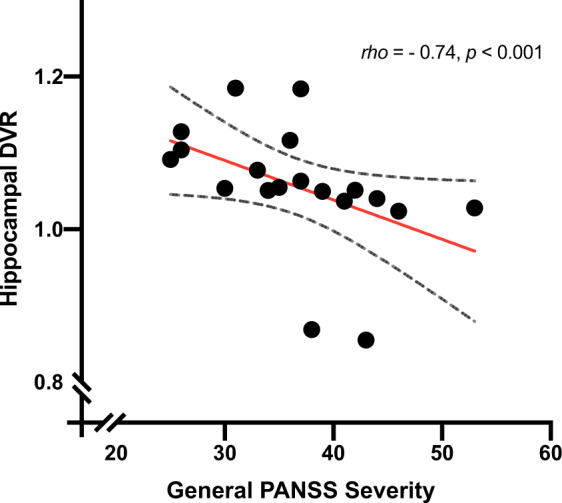
Relationship of tracer uptake in the hippocampus, as measured by DVR, and General PANSS symptom severity. Dashed line represents 95% CI.

**Fig. 5:**
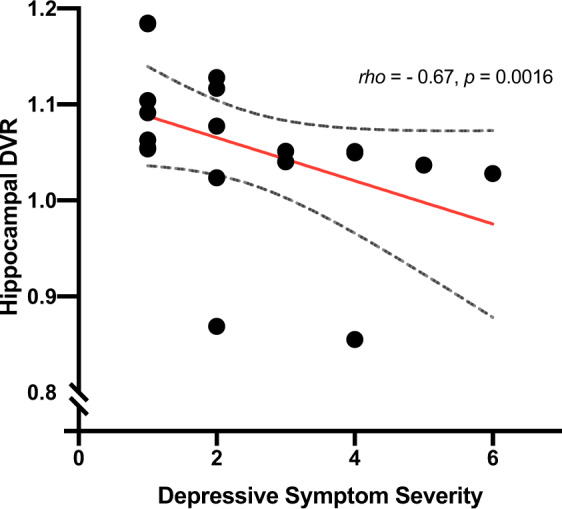
Relationship of tracer uptake in the hippocampus, as measured by DVR, and depression symptom severity. Dashed line represents 95% CI.

There was a significant negative association between PANSS total symptom severity and hippocampal DVR (rho = −0.47, *p* = 0.04; Fig. 3). In addition, there was a significant negative association between hippocampal DVR and PANSS general symptom severity (rho = −0.74, *p* < 0.001; Fig. 4), but not with positive (rho = −0.41, *p* = 0.08), or negative (rho = 0.35, *p* = 0.14) symptom severity. Hippocampal DVR was also negatively associated with the depression item of the PANSS scale (rho = −0.67, *p* = 0.002; Fig. 5). The negative correlation between both general symptom severity and depression item with hippocampal DVR survived Bonferroni correction (*α*_corrected_ = 0.05/5 = 0.01).

The association between hippocampal *V*_T_, and PANSS scores, was assessed using Pearson’s correlation. The relationship between *V*_T_ and total PANSS was not significant (*r* = −0.43, *p* = 0.06; eFigure 3 in Supplementary Material), along with positive PANSS (*r* = −0.30, *p* = 0.21), and negative PANSS (*r* = 0.08, *p* = 0.76). However, there was a significant negative correlation between hippocampal *V*_T_ and general PANSS (*r* = −0.53, *p* = 0.02, eFigure 4 in Supplementary Material), and the depression item of the PANSS (*r* = −0.56, *p* = 0.01, eFigure5 in Supplementary Material).

## Discussion

We found a significantly lower DVR of the NMDAR selective radiotracer, [^18^F]GE-179, in the hippocampus in patients with schizophrenia compared to healthy controls. In absolute terms, the hippocampal *V*_T_ values of [^18^F]GE-179 were also lower in schizophrenia compared to controls, but this difference was not statistically significant. *V*_T_ values showed more than double the variability seen with DVR (coefficients of variation for the hippocampus in healthy controls were 18.2% and 7.1%, respectively). Thus, the greater noise in the *V*_T_ measure may have contributed to the lack of a significant difference in this measure in the hippocampus compared to the DVR.

This is the first in vivo study using an NMDAR selective radiotracer in patients with first-episode schizophrenia and builds upon the one previous in vivo study of NMDAR availability that used a SPECT tracer in older patients with chronic schizophrenia (illness duration mean (S.D) = 15.23 (9.21) years) [19]. In keeping with the present findings, they found a significant reduction in the DVR of [^123^I]CNS-1261 in the left hippocampus relative to the whole brain in medication-free patients (*n* = 5) compared to healthy controls. Our study findings are consistent with these, and, importantly, extend them to first-episode patients, indicating that alterations in NMDAR availability in the hippocampus are evident early in the development of the disorder. However, similar to our study, the SPECT study did not find a difference in NMDAR availability as measured by the *V*_T_ in patients relative to healthy controls, although they did report a global reduction in [^123^I]CNS-1261 *V*_T_ in patients treated with clozapine (*n* = 9) [20].

Our findings are also consistent with post-mortem data which provide evidence for lower hippocampal availability of NMDAR in patients with schizophrenia measured using MK801 binding [37], and lower hippocampal NR1 mRNA and protein levels in the hippocampus in schizophrenia patients relative to healthy controls [38]. As the NR1 subunits are essential components of the NMDAR, they can be used as a measure of NMDAR number more reliably than measurements of other subunits [39].

### Strengths and limitations

A strength of our study is that the patients were predominantly antipsychotic-free FEP patients, and the sample size was more than double that of the SPECT study in chronic patients [19]. One important consideration is that the *V*_T_ and DVR do not differentiate between specific and non-specific binding. As such, we cannot exclude the possibility that there was an alteration in non-specific binding contributing to the group differences in DVR. [^18^F]GE-179 has nanomolar affinity for the PCP site of the NMDAR ligand-gated ion channel and low affinity for other CNS receptors, indicating that its binding is likely to largely reflect NMDAR [40]. However, in vivo studies have been mixed. A study that used a blocking agent to investigate the specific binding of [^18^F]GE-179 in rodents and primates did not find evidence of specificity to NMDAR [41]. However, this study co-administered anaesthetic agents which may also alter the availability of the target sites, complicating interpretation of the findings as tracer binding could have been altered by anaesthetic agents used in the control group [41–43]. Notwithstanding this, a recent study found that electrical stimulation designed to activate NMDAR induced significant increases in the uptake of [^18^F]GE-179, and also that the NMDAR antagonist, ketamine, blocked this increase in uptake, indicating [^18^F]GE-179 binds to NMDAR and is sensitive to changes in NMDAR activity [44]. This provides evidence that the tracer we used is specific to the NMDAR, and sensitive to manipulations that activate NMDAR. However, it should be noted that in contrast to this study, in our study subjects were at rest. Future studies using activation paradigms or cognitive tasks would be useful to investigate the impact of receptor activation on specific binding in patients. There have also been studies in awake animals and humans using similar arylguanidine-based ligands to the one we used, such as CNS 5161 [45] and GMOM [46]. These studies have shown reductions in tracer uptake by NMDAR binding site antagonists. Using ketamine to block binding to the NMDAR, Van der Doef et al. reported a reduction in mean [^11^C]GMOM inhibitory constant (K_i_) of 70% +/−12 in the hippocampus, indicating specific binding in vivo in the absence of the use of an NMDAR-modulating anaesthetic [46]. While these studies suggest that a large proportion of our signal is likely to be specific to the NMDAR, blocking studies in humans are required to determine how much of [^18^F]GE-179 tracer uptake is specific to the NMDAR in patients. However, this will be challenging because it would require giving patients a substantial dose of an NMDAR blocker such as ketamine, which can induce potent psychoactive effects in patients, which participants may find hard to tolerate while being scanned.

It is important to recognize that partial volume effects can potentially contribute to the lower hippocampal DVR values in patients. However, hippocampal and whole-brain volumes were not significantly different between the groups, and there was no correlation between hippocampal DVR and hippocampal volume; nor did our results change when hippocampal volume was added into the analysis as a covariate. These analyses suggest that volume effects are not a major factor in our finding of lower hippocampal DVR. There were also no significant differences in cumulative movement between patients and controls, indicating that partial volume effects or excess movement are unlikely to be major contributors to the group differences in DVR. It is possible that our findings were influenced by environmental risk factors for psychosis as we did not match our groups based on parental socio-economic status.

### Interpretation and implications for understanding the pathophysiology of psychosis

As DVR is relative to tracer uptake in the brain, it is also possible that lower hippocampal DVR reflects greater uptake in other brain regions in schizophrenia. However, if this were the case then *V*_T_ values would be higher in other brain regions in schizophrenia, which was not the case, and there is no consistent post-mortem evidence of globally increased NMDAR levels in schizophrenia [18]. Indeed, post-mortem studies in schizophrenia show lower brain levels of NMDAR in the hippocampus [18], cortex [47–49] and thalamus [50, 51] relative to controls. Thus, taken together with post-mortem findings of lower NMDAR levels [37, 38] the most parsimonious explanation for our findings is that first-episode patients with schizophrenia have lower hippocampal NMDAR density compared to healthy volunteers. However, as [^18^F]GE-179 binds to the NMDAR intrachannel phencyclidine binding site when the channel is open, another possibility is that a reduction in channel opening/receptor activity contributed to our results [21]. Recently, [^18^F]GE-179 has shown to have increased uptake following deep brain stimulation (DBS) of the hippocampus, without significant changes in cerebral blood flow, showing that the tracer is sensitive to a stimulus that leads to NMDAR activation [40]. Notwithstanding this, in either case the net result would be lower hippocampal NMDAR signalling, consistent with hypotheses that there is NMDA hypofunction in schizophrenia, which would be expected to alter excitation-inhibition balance to impair hippocampal function [52]. Our findings also add to other evidence for the involvement of the hippocampus in the pathophysiology of schizophrenia [53–57].

When we conducted exploratory subgroup analyses, we found that, there was no significant difference in hippocampal DVR between antipsychotic-free patients and healthy controls, although DVR was lower in absolute terms in patients. In contrast, the antipsychotic-treated patients had significantly lower hippocampal DVR compared to healthy controls. It should be recognized that these analyses are exploratory and the study was not designed or powered to detect differences in subgroups. Nevertheless, this could suggest either an effect of antipsychotic treatment on NMDAR or that patient subgroups show differences in NMDAR availability. The antipsychotics used by our patient group have very low affinities (K_i_ >1000 nM) for the tracer’s binding site on the NMDAR (the PCP binding site: Aripiprazole K_i_ >4000 nM, Olanzapine K_i_ >10,000 nM, Risperidone K_i_ >10,000 nM) [58] (https://pdsp.unc.edu/databases/kidb.php). There are no data on the K_i_ for Lurasidone on NMDAR but there is evidence that Lurasidone only has a weak affinity for NMDAR [59] and only one patient was treated with this. Furthermore, there was no correlation between antipsychotic dose and hippocampal DVR. Thus, it is unlikely that the results we see are due to the direct action of the medication on the receptor. All the patients treated with antipsychotics continued to be symptomatic despite adequate treatment perhaps suggesting a poor response to dopamine targeting medications. There is evidence that poor treatment responders have less significant abnormalities in their dopamine system and greater glutamate dysfunction [28, 60–62]. Our finding may, thus, suggest that NMDAR abnormalities are more marked in patients who show poor response to antipsychotic treatment. It would be useful to test this in a future study.

There was a negative association between hippocampal DVR and PANSS total symptoms, in agreement with prior findings in chronic patients treated with antipsychotic medication [19]. We found no significant associations with positive or negative subscales, in contrast to Pilowsky et al. who found a negative association with negative symptom severity. We cannot exclude the possibility that there is a weaker relationship with these symptoms. Nevertheless, this finding indicates that hippocampal NMDAR alterations may not be directly linked to psychotic symptoms, consistent with models that these arise from hyperdopaminergia [5, 63], albeit this may be secondary to NMDAR dysfunction in the hippocampus [64–67]. Preclinical models of psychosis suggest that hippocampal NMDAR hypofunction results in increased glutamatergic activity in pathways that project from the hippocampus to lead to increased mesostriatal dopaminergic activity, which is thought to result in psychosis [68, 69]. Interactions between the NMDAR and serotonin system may also contribute to psychosis [67].

It is also possible that glutamate dysfunction in other areas has a greater role in the induction of symptoms. There is evidence to suggest that glutamate levels in the ACC are related to greater severity of psychotic symptoms at first presentation [70]. Furthermore, a recent study found that thalamic glutamate levels in antipsychotic-naive patients were significantly negatively correlated with a percent change in symptoms after antipsychotic treatment [62].

The lack of a strong direct relationship between DVR and psychotic symptom severity may explain why it has proven challenging to develop antipsychotic medications targeting the NMDAR, such as glycine site modulators [71], inhibitors of glycine reuptake [72], positive allosteric modulation of NMDAR subunits [73], and other aspects of glutamatergic function for schizophrenia [73]. We found a significant inverse association between DVR and the general symptom subscale, which includes items covering cognitive symptoms. Preclinical studies show hippocampal NMDAR plays a key role in learning and memory [74, 75]. There is also evidence that NMDAR blockers, such as ketamine, induce cognitive impairments in patients with psychosis and healthy controls, and cognitive impairments are seen in chronic ketamine users [76–79]. Furthermore, there is evidence that patients with schizophrenia have impairments in cortical plasticity which can be improved with NMDAR enhancers such as D-serine [80]. These lines of evidence indicate that lower NMDAR could contribute to cognitive impairments in schizophrenia. However, further work is needed to determine if lower NMDAR availability is associated with cognitive impairments. Moreover, our exploratory analysis also demonstrated an inverse relationship between PANSS depressive symptom severity and hippocampal *V*_T_ and DVR of [^18^F]GE-179, extending a prior finding that NMDAR tracer binding was globally reduced in patients with epilepsy who received treatment for depression [21], to show a direct association with depressive symptoms for the first time, as far as we are aware. This finding also adds to other evidence indicating a role for NMDAR in the pathophysiology of depressive symptoms, potentially more generally than just in schizophrenia. However, it is not clear how this fits with findings that treatment with NMDAR antagonist drugs, such as esketamine and ketamine, induces rapid antidepressant effects [81, 82]. A future study in patients with major depressive disorders would be useful to test this.

### Conclusions

Our findings indicate that patients with first-episode psychosis show lower hippocampal NMDAR availability relative to other brain regions. This is consistent with patients showing either lower activity and/or density of NMDAR in the hippocampus relative to the whole brain; and the hypothesis that NMDA hypofunction in the hippocampus plays a key role in the pathophysiology of schizophrenia. Furthermore, we find that lower relative hippocampal NMDAR availability is associated with greater total symptom severity. Further studies are needed to determine the role of the NMDAR in the development of symptoms.

## Supplementary information

Supplementary
