# Supplementary material for: N-methyl-D-aspartate receptor availability in first-episode psychosis: a PET-MR brain imaging study
Source: Transl Psychiatry. 2021 Aug 12;11:425. doi: 10.1038/s41398-021-01540-2 (PMC8361127; doi:10.1038/s41398-021-01540-2)
Supplement: Supplementary file 1 — Supplementary [file 41398_2021_1540_MOESM1_ESM.docx]

**N-Methyl-D-Aspartate Receptor availability in First-Episode Psychosis: A PET-MR brain imaging study**

**eMethods 1:** Full Exclusion Criteria

**eMethods 2:** PET Imaging Acquisition Parameters

**eMethods 3:** MRI Imaging Acquisition Parameters

**eMethods 4:** PET Image Analysis: Preprocessing Methods

**eMethods 5:** [^18^F]GE-179 Uptake: Kinetic Modelling Validation

**eMethods 6:** Movement Parameters

**eMethods 7:** Volumetric Image Analysis

**eMethods 8**: [^18^F]GE-179 Uptake: Additional Analyses

**eMethods 9:** Voxel-wise Analysis: Additional Analyses

**eTables 1:** PET data

**eTables 2:** Experimental Parameters

**eResults 1:** Volumetric Image Analysis

**eResults 2:** [^18^F]GE-179 Uptake: Additional Region-of -Interest Analysis

**eResults 3:** [^18^F]GE-179 Uptake: Voxel-wise Analysis

**eFigure 1:** Hippocampal DVR subgroup analysis

**eFigure 2:** Hippocampal V_T_ subgroup analysis

**eFigures 3-5:** Hippocampal [^18^F]GE-179 V_T_ and symptoms

**eReferences**

**eMethods 1**. Full Exclusion Criteria

Exclusion criteria for all participants: 1) ages <18 or >40; 2) a history of a head injury resulting in loss of consciousness; 3) personal history of serious medical illness; 4) contraindications to MRI or PET safety; 5) current or lifetime history of substance use or dependence as determined by the Structured Clinical Interview for DSM-IV-TR (SCID-I/P); 6) screened positive for any of the following substances (except cannabis) on a multi-panel urine drug screen detecting the following substance; amphetamine (300ng/ml cut off), cocaine (150 ng/ml cut off), ketamine (1000 ng/ml cut off), cannabis (50ng/ml cut off), methamphetamine (300 ng/ml cut off), opiates (2000 ng/ml cut off) (SureScreen Diagnostics, Derby); 7) Current use of medications that might affect the glutamate system (antidepressants, benzodiazapines, mood stablizers)

**eMethods 2.** PET Imaging Acquisition Parameters

All participants underwent a dynamic, continuous 90 minutes simultaneous PET-MR scan. All scans took place at the same time, in the early afternoon (13:00-15:00). PET scans were acquired in list mode for 90 minutes using a Siemens 3T Magnetom Biograph mMR PET/MR hybrid scanner (Siemens, Erlangen, Germany). At the beginning of the scan, a bolus of [^18^F]GE-179 was manually injected over ~10 seconds, followed by a 10mL saline flush of the syringe and extension line.

At the completion of the acquisition, a separate low dose CT scan (140 kV, 10 mA, helical acquisition) of the subject’s head containing the complete mMR field of view was acquired on a GE Discovery DST 710 PET-CT. This was registered to the structural T1-weighted MP-RAGE MRI (see eMethods 3) to create a CT-based µ-map for attenuation correction.

Attenuation corrected dynamic PET data were reconstructed using Ordered Subset Expectation Maximisation (OSEM) (2 subsets, 21 iterations) to 28 frames of increasing duration (8 x 15 sec, 3 x 60 sec, 5 x 120 sec, 9 x 300 sec, 3 x 600 sec). During reconstruction PET data were also corrected for random and scatter coincidences.

### In parallel to the PET/MRI acquisition, continuous arterial sampling using an MR compatible blood sampler (<http://www.swisstrace.ch/blood-sampler-twilite.html)> was performed for the first 16 minutes followed by 6 discrete samples (20, 30, 45, 60, 75 and 90 minutes post injection). This was done to obtain individualised parent plasma input functions for each participant after correction for the radioactivity in blood cells and radioactive metabolites. To determine the plasma tracer radioactivity and to correct for the radiolabeled metabolites the plasma-over-blood (POB = concentration radiotracer in plasma/ concentration radiotracer in whole blood) and the parent plasma fraction (PPf = fraction of authentic radiotracer in plasma over the total plasma radioactivity) were defined for each PET acquisition.

Arterial blood samples were collected in dipotassium EDTA lined blood tubes (BD #367873) at specific time intervals. 200 μL of blood was removed for gamma counting. The remainder was centrifuged at ~ 3000 g at room temperature (RT) for 5 minutes to separate the plasma. 200 μL of plasma was also removed for gamma counting. The remaining plasma was mixed with an equal volume of acetonitrile, and vortexed before being centrifuged again at ~3000g at RT for 5 minutes. 1 mL of the supernatant was injected into a high-performance liquid chromatography (HPLC) system (Agilent 1200 series) to separate and quantify the parent radiotracer from metabolites. A semi-preparative Luna® 10 μm C18(2) 100 Å, 250 × 10 mm column (Phenomenex# 00G-4253-N0) was used. An isocratic gradient of 50% solvent A (acetonitrile) 50% solvent B (70 mM NaH_2_PO_4_) at a flow rate of 5 mL/min was applied and 1 mL fractions were collected for the duration of the run. HPLC detection was carried out using a serial UV detector (λ = 254 nm) and the collected fractions were counted on a calibrated gamma-counter (Wizard2 2470, Perkin-Elmer).

**eMethods 3:** MRI Imaging Acquisition Parameters

High-resolution 3D T1-weighted images were acquired for anatomical co-localisation on a Siemens Biograph mMR 3T using MP-RAGE (matrix: 224 x 256, FoV – 270mm) with the following scan parameters: repetition time: 1700 msec, echo time: 2.63 msec, inversion time: 900msec, flip angle: 9°, slice thickness: 1.10 mm, number partitions: 176)

**eMethods 4:** PET Image Analysis: Preprocessing Methods

### *Data pre-processing*

Data pre-processing was performed using a combination of Statistical Parametric Mapping 12 (<http://www.fil.ion.ucl.ac.uk/spm>) and FSL (<http://www.fsl.fmrib.ox.ac.uk/fsl>) functions, as implemented in MIAKAT (<http://www.imanova.co.uk)>. Motion correction was applied for all PET scans.

Attenuated corrected frames were realigned to a single “reference” frame, by employing a mutual information algorithm, creating a movement-corrected dynamic image, which was then used in the analysis. Realigned frames were then summated to create an individual motion-corrected reference map for the brain tissue segmentation. Specifically, individual T1-weighted MR images were co-registered to the PET image using rigid body transformation. Normalisation parameters were obtained by warping the co-registered structural MRI to MNI space (International Consortium for Brain Mapping ICBM/MNI) using bias-corrected segmentation in SPM12. The inverse of these parameters was used to fit a neuroanatomical atlas (Hammersmith Atlas 83 ROIs: <http://brain-development.org/brain-atlases/adult-brain-maximum-probability-map-hammers-mith-atlas-n30r83-in-mni-space)> to each individual PET scan. ^1,2^ Of the available ROIs, a subset of 5 regions were considered, including cortical and subcortical regions. For bilateral regions, left and right hemispheres were analyzed in combination.

*Blood data processing*

Blood input functions (both whole blood data and plasma data), POB and PPf modelling were performed using MultiBlood, a unified framework for the arterial data modelling to achieve an accurate and fully-automated description of the plasma tracer kinetics.^3^ The pipeline employed basis pursuit techniques for estimating both radiometabolites and parent concentration models from the raw plasma measurements, allowing the resulting algorithm to be both robust and flexible to the different quality of data available.^4^ This pipeline was validated for the analysis of blood data obtained from MR-compatible samplers, showing that this method can filter out the excess noise and produce blood data time-activity courses comparable to traditional blood samplers.^5^

*Kinetic analysis*

For the region-of-interest analysis, we implemented the standard two-tissue compartmental model, consistent with previous analysis indicating it as the best model to describe [^18^F]GE179 brain kinetics.^6^ Identification of model parameters were done using nonlinear estimator (matlab lsqnonlin.m) and weighting each data point for the inverse of its error variance. The blood volume parameter (Vb) was fixed at 5% to reduce the error induced by the noisy blood input functions into the parameter estimates.

We also implemented Logan graphical plot^7^ and multilinear regression analysis^8^ for comparative reasons.

The region-of-interest analysis was conducted blind to patient and medication status. Our primary endpoint was [^18^F]GE-179 uptake for hippocampus. This area was chosen because of evidence suggesting its role in the pathophysiology of schizophrenia^9^ and possible dysfunction of glutamate in these areas.^10^ ^11^ The ROIs were obtained from the Hammersmith atlas, a standard probabilistic neuroanatomical atlas.^2^ Using Statistical Parametric Mapping 12 (SPM12; version 6684)^12^ gray matter masks were obtained by binarising segmented gray matter from T1-weighted images and applying this to the Hammersmith atlas.^2^

*Quality control*

The outputs of the image analysis were manually controlled for by two experienced PET modelers (MV and BS) blind to subject group. Specifically we tested that 1) brain extraction did not include loss of brain or excess of non-brain tissue, 2) GM, WM and CSF were properly extracted, 3) both MNI structural template and Hammersmith atlas were aligned to individual MRI, 4) PET frames were realigned to the same space correcting for subject inter-frame motion, 5) the fitting of the blood input function provided by Multiblood was physiological, 6) the fitting of brain PET data provided by kinetic modelling was physiological, and 7) the coefficient of variation (CV) for V_T_ estimates were lower than 20% for each ROI. Those scans failing any of point 1 to 6 were labelled as QC failure and excluded from the analysis.

**eMethods 5:** [^18^F]GE-179 Uptake: Kinetic Modelling Validation

We compared the V_T_ results obtained from 2TCM with those from graphical analysis (both Logan and MA1). Logan V_T_ estimates were all highly correlated with 2TCM V_T_ estimates (Pearson’s correlation ranging from r=0.80 in occipital cortex up to r=0.99 in parietal cortex). The mean relative difference was 2%±1%. MA1 V_T_ estimates were also all highly correlated with 2TCM V_T_ estimates (Pearson’s correlation ranging from r=0.80 in occipital cortex up to r=0.99 in thalamus). The mean relative difference was 3%±1%.

**eMethods 6:** Movement Parameters

Cumulative movement was defined the sum of frame-by-frame Euclidean distance, calculated by the frame realignment during motion correction.

**eMethods 7:** Volumetric Image Analysis

To identify if there were volumetric differences between patients and controls, whole-brain and hippocampal volumes were compared using independent t-tests. ROI volumes were extracted from the atlas-based segmentation of the PET and MRI images. Briefly, the MRI template was nonlinearly registered to the subject’s brain MRI and the resulting deformation field saved. The latter was hence applied to the Hammersmith atlas to provide a brain individual anatomical segmentation for both PET and MRI images. These steps were implemented in the MIAKAT as part of its standard image pre-processing.

**eMethods 8**: [^18^F]GE-179 Uptake: Additional Analyses

To determine whether the volume of distribution or DVR was lower in patients in other brain regions than our primary region of interest, a repeated measures ANOVA using a 2 (group) x 4 (ROI) design was used to determine if other areas (Anterior Cingulate Cortex (ACC), thalamus, striatum, and temporal lobe) known to be involved in schizophrenia had lower [^18^F]GE-179 receptor availability. These regions of interested were defined using the Hammersmith atlas.^2^

Due to poor quality of the data 2 individual data sets were removed for the ACC ROI analysis and 1 data set was removed from the thalamus ROI analysis leaving 17 healthy controls and 18 patients, of which 11 were antipsychotic-free, and 17 healthy controls and 19 patients, of which 11 were antipsychotic-free, for each analysis respectively.

**eMethods 9:** Voxel-wise Analysis: Additional Analyses

**Whole-brain analysis**
An exploratory, whole-brain, voxel-wise analysis was conducted using SPM12. An independent samples t-test was used to investigate if there were whole-brain voxel-wise differences in the volume of distribution (V_T_) of [^18^F]GE-179 in whole-brain gray matter between patients and healthy volunteers. The height threshold was set to p=0.001 and peak-level family-wise error corrected thresholds (p<0.05) were used. However, the results of this analysis were also explored using a liberal, uncorrected threshold (p<0.001).

**Region of interest analysis**

An exploratory, ROI analysis of the hippocampus was also conducted using SPM12. An independent samples t-test was used to investigate if there were regional differences in the V_T_ of [^18^F]GE-179 in the hippocampus between patients and healthy volunteers. The hippocampal mask was defined using the Hammersmith Atlas.^2^ The height threshold was set to p=0.001 and peak-level family-wise error corrected thresholds (p<0.05) were used. However, the results of this analysis were also explored using a liberal, uncorrected threshold (p<0.001).

**eTable 1**. PET data

|  | Healthy volunteers/  Mean (SD) | Patients with FEP/  Mean (SD) | Healthy Volunteers’ Coefficient of Variation (%) | Patients’ Coefficient of Variation (%) | t | df | p |
| --- | --- | --- | --- | --- | --- | --- | --- |
| Hippocampal volumes (mm^3^) | 4480.44 (445.70) | 4530.53 (359.09) | 9.9 | 7.9 | -0.38 | 35 | 0.71 |
| Whole brain volumes (mm^3^) | 1251278.22  (123971.47) | 1253046.74 (103049.00) | 9.9 | 8.2 | -0.05 | 35 | 0.96 |
| Hippocampal V_T_ (ml/cm^3^) | 9.67 (1.76) | 8.75 (1.99) | 18.2 | 22.7 | 1.49 | 35 | 0.15 |
| Thalamus V_T_  (ml/cm^3^) | 11.17 (1.96) | 10.23 (2.32) | 17.5 | 22.7 | 1.22 | 34 | 0.23 |
| ACC V_T_  (ml/cm^3^) | 8.74 (1.35) | 8.50 (2.15) | 15.4 | 25.3 | 0.39 | 33 | 0.70 |
| Striatum V_T_  (ml/cm^3^) | 9.48 (1.78) | 9.27 (2.05) | 18.8 | 22.1 | 0.32 | 35 | 0.75 |
| Temporal lobe V_T_ (ml/cm^3^) | 8.74 (1.42) | 8.50 (1.97) | 16.2 | 23.2 | 0.43 | 35 | 0.67 |
| Hippocampal DVR | 1.12 (0.08) | 1.06 (0.08) | 7.1 | 7.5 | Mann-Whitney U = 92.00 |  | 0.02 |
| Thalamus DVR | 1.27 (0.06) | 1.24 (0.07) | 4.7 | 5.6 | Mann-Whitney U = 146.00 |  | 0.64 |
| ACC DVR | 1.00 (0.05) | 1.02 (0.10) | 5.0 | 9.8 | Mann-Whitney U = 175.00 |  | 0.48 |
| Striatum DVR | 1.10 (0.05) | 1.12 (0.08) | 4.5 | 7.1 | Mann-Whitney U = 192.00 |  | 0.54 |
| Temporal lobe DVR | 1.02 (0.02) | 1.03 (0.13) | 2.0 | 12.6 | Mann-Whitney U = 142.00 |  | 0.39 |

ACC – anterior cingulate cortex. V_T_ – volume of distribution. DVR – distribution volume ratio.

**eTable 2**. Experimental Parameters

|  | Healthy volunteers/  Mean (SD) | Patients with FEP/  Mean (SD) | Mann-Whitney U | df | p |
| --- | --- | --- | --- | --- | --- |
| N | 18 | 19 |  |  |  |
| Weight (Kg) | 75.62 (13.02) | 79.05 (15.01) | 133.50 |  | 0.26 |
| Body Mass Index | 24.38 (3.13) | 26.02 (3.65) | t = -1.48 * | 35 | 0.15 |
| Dose (Mbq) | 141.03 (7 .15) | 139.63 (10.68) | 139.00 |  | 0.34 |
| Molar activity GBq/μmol | 66.19 (36.98) | 86.84 (67.89) | 157.50 |  | 0.69 |
| Injected mass (μg) | 1.04 (0.56) | 1.08 (0.86) | 160.50 |  | 0.75 |
| Total motion during 90min scan (mm) | 6.99 (4.31) | 12.63 (10.07) | 119.00 |  | 0.12 |
| Input function: AUC start (5mins) | 2.89 (1.13) | 2.68 (1.29) | 134.00 |  | 0.27 |
| Input function: AUC end | 4.19 (1.32) | 4.31 (1.88) | 153.00 |  | 0.60 |
| Input function:  AUC total | 7.09 (2.36) | 6.99 (3.11) | 139.00 |  | 0.34 |

AUC – area under the curve. *Independent samples t-test

**eResults 1**. Volumetric Image Analysis

To identify if there were volumetric differences between patients and controls, tissue volumes were compared between patients and controls in whole-brain and region of interest analyses of the hippocampus. There were no significant group differences in tissue volumes between patients and controls (t_35_ = -0.047, p = 0.96 ) in whole-brain and region of interest analyses if of the hippocampus (t_35_ = -0.38, p = 0.71 ), ACC (t_35_ = -0.46, p = 0.65), thalamus (U = 132, p = 0.25), striatum (U = 170, p = 0.99 ), temporal lobe (t_35_ = -0.41, p = 0.69).

**eResults 2.** [^18^F]GE-179 Uptake: Additional Region-of -Interest Analysis

To determine if [^18^F]GE-179 uptake was lower in patients across additional brain regions for comparison with future studies, independent *t*-tests were performed for V_T_ analysis as this data set was normally distributed. For the non-normally distributed DVR data, Mann-Whitney tests were performed to determine if other areas known to be involved in schizophrenia had lower NMDAR availaiblity. These regions of interested were defined using the Hammersmith atlas.^2^

**Result:**

**V_T_ Analysis:**

*Patients v Healthy controls:*

For this analysis there were 16 healthy controls and 18 patients included in the final analysis.

The results show that there was a statistically non-significant effect of group on ROI NMDAR availability (F(1, 32) = 1.23, p = 0.28).

Separate independent sample t-tests on the outcome variables found that there was not a significant effect of group on V_T_ in the ACC (t_33_ = 0.39, p = 0.70, Cohen’s d = 0.13), thalamus (t_34_ = 1.22, p = 0.23, Cohen’s d = 0.41), striatum (t_35_ 0.32, p = 0.75, Cohen’s d = 0.11), or temporal lobe (t_35_ = 0.43, p = 0.67, Cohen’s d = 0.14).

In addition, there was no significant effect of group on V_T_ in the whole brain when the full sample was used (t_35_ 0.60, p = 0.56, Cohen’s d = 0.20).

**DVR Analysis:**

*Patients v Healthy controls:*

For this analysis there were 16 healthy controls and 18 patients included in the final analysis. Non-parametric analysis techniques (namely Mann-Whitney U) were used, as the spread of DVR data was not normally distributed.

Analysis with independent sample Mann-Whitney U test found that there was no significant effect of group on DVR in the ACC (U = 175.00 , z = 0.726 , p = 0.483, Cohen’s d = 0.19), thalamus (U= 146.00 , z = -0.491 , p = 0.639, Cohen’s d = 0.41), striatum (U = 192.00, z = 0.638, p = 0.538, Cohen’s d = 0.31), or temporal lobe (U = 142.00, z = -0.881, p = 0.391, Cohen’s d = 0.12).

**eResults 3.** [^18^F]GE-179 Uptake: Voxel-wise Analysis

**Whole-brain analysis**

A whole-brain voxel-wise analysis showed that there were no significant differences between patients relative to controls in the V_T_ of [^18^F]GE-179 when using family-wise error corrected thresholds (p<0.05). An exploratory analysis showed that there were also no significant differences between patients relative to controls in the VT of [^18^F]GE-179 when using liberal uncorrected thresholds (p<0.001).

**Region of interest analysis**

A region of interest analysis of the hippocampus showed that there were no significant differences between patients relative to controls in the VT of [^18^F]GE-179 when using family-wise error corrected thresholds (p<0.05). An exploratory analysis showed that there were also no significant differences between patients relative to controls in the VT of [^18^F]GE-179 in the hippocampus when liberal, uncorrected thresholds were used (p<0.001).


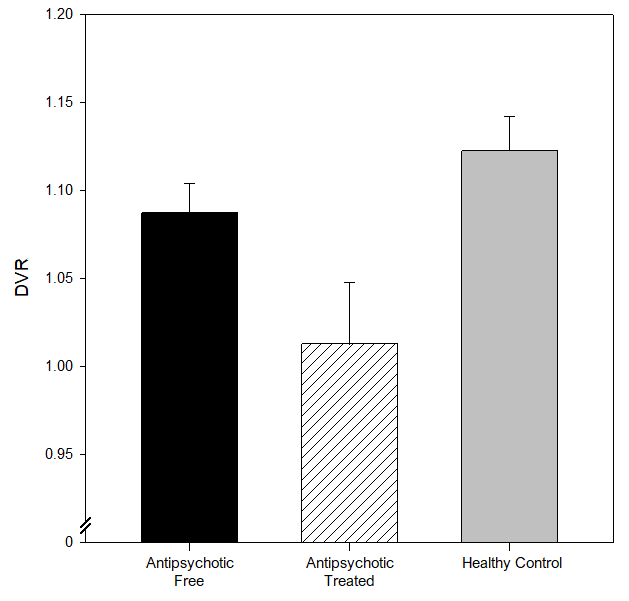


**eFigure 1: Hippocampal DVR subgroup analysis.** NMDAR availability in the hippocampus measured by positron emission tomographic (PET) imaging. Data are expressed as mean (SE) of the distribution volume ratio (DVR) of [^18^F]GE-179.


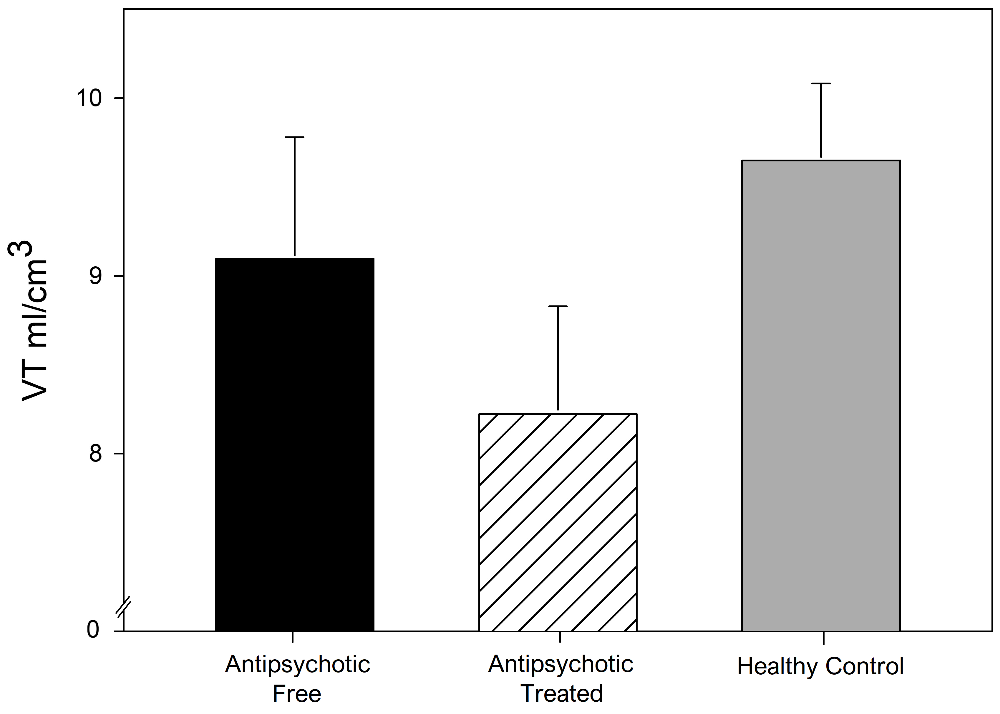


**eFigure 2: Hippocampal V_T_ subgroup analysis.** NMDAR availability in the hippocampus measured by positron emission tomographic (PET) imaging. Data are expressed as mean (SE) of the distribution volume (V_T_) of [^18^F]GE-179.

**
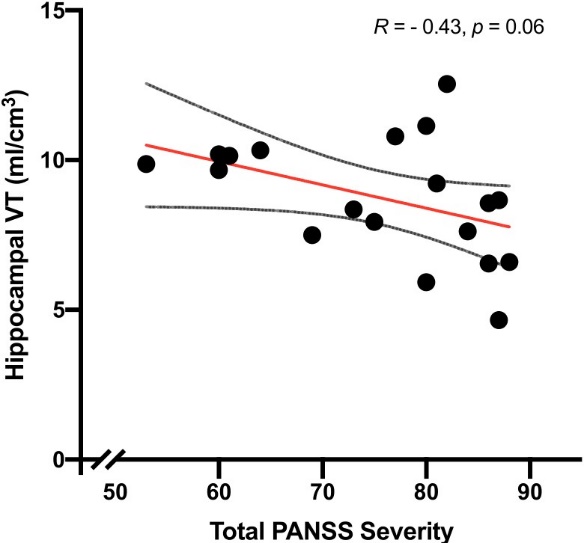
**

**eFigure 3: Relationship of NMDAR availability as measured by V_T_ in the hippocampus and Total PANSS symptom severity.** Dashed line represents 95% CI.

**
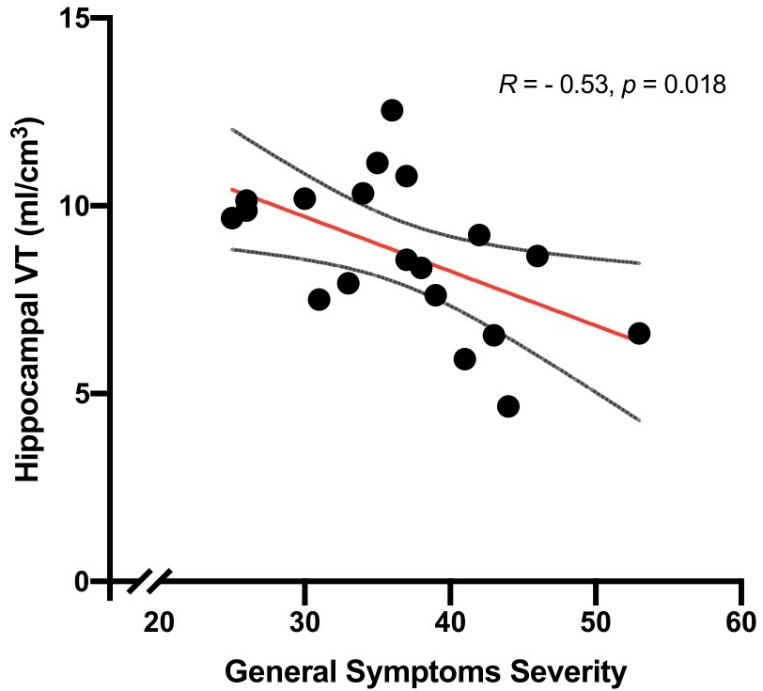
**

**eFigure 4:** **Relationship of NMDAR availability as measured by V_T_ in the hippocampus and General PANSS symptom severity.** Dashed line represents 95% CI.

**
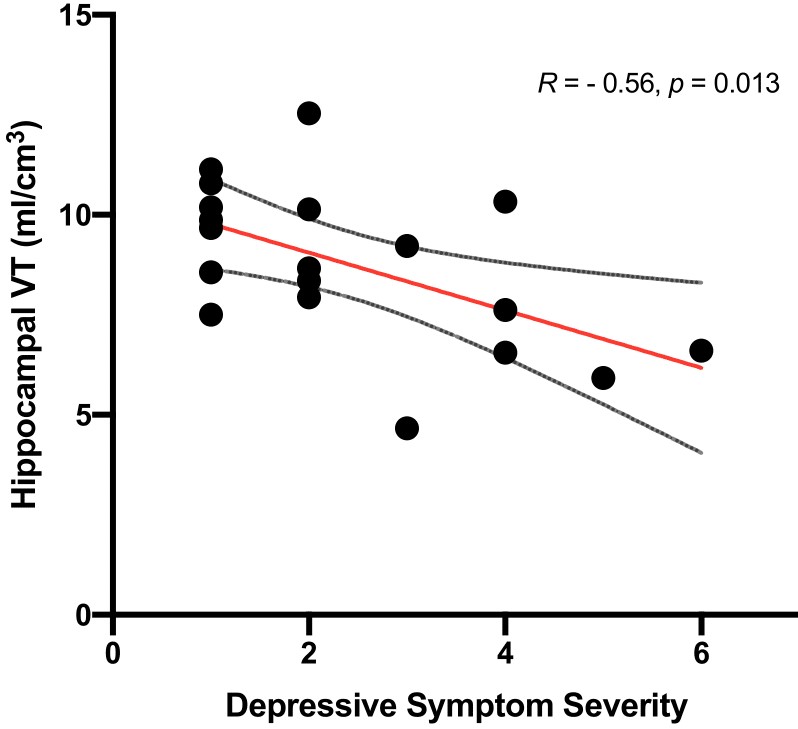
**

**eFigure 5: Relationship of NMDAR availability as measured by VT in the hippocampus and depression symptom severity.** Dashed line represents 95% CI.

**eReferences**

1. Gousias IS, Rueckert D, Heckemann RA, Dyet LE, Boardman JP, Edwards AD, et al. Automatic segmentation of brain MRIs of 2-year-olds into 83 regions of interest. Neuroimage. 2008 Apr;40(**2**):672–84.

2. Hammers A, Allom R, Koepp MJ, Free SL, Myers R, Lemieux L, et al. Three-dimensional maximum probability atlas of the human brain, with particular reference to the temporal lobe. Hum Brain Mapp. 2003 Aug;19(**4**):224–47.

3. Tonietto M, Veronese M, Rizzo G, Zanotti-Fregonara P, Lohith TG, Fujita M, et al. Improved models for plasma radiometabolite correction and their impact on kinetic quantification in PET studies. J Cereb Blood Flow Metab. 2015 Sep;35(**9**):1462–9.

4. Tonietto M, Rizzo G, Veronese M, Borgan F, Bloomfield PS, Howes O, et al. A unified framework for plasma data modeling in dynamic positron emission tomography studies. IEEE Trans Biomed Eng. 2019 May;66(**5**):1447–55.

5. Santangelo B, Dunn J, Beck K, McGinnity CJ, Tonietto M, Turkheimer F, et al. Modelling continuous arterial blood data from MR-compatible sampler in simultenous PET-MRI experiments. In: 2019 IEEE 16th International Symposium on Biomedical Imaging (ISBI 2019). IEEE; 2019. p. 750–3.

6. McGinnity CJ, Hammers A, Riaño Barros DA, Luthra SK, Jones PA, Trigg W, et al. Initial evaluation of 18F-GE-179, a putative PET Tracer for activated N-methyl D-aspartate receptors. J Nucl Med. 2014 Mar;55(**3**):423–30.

7. Logan J, Fowler JS, Volkow ND, Wolf AP, Dewey SL, Schlyer DJ, et al. Graphical analysis of reversible radioligand binding from time—activity measurements applied to [ *N* - ^11^ C-Methyl]-(−)-cocaine PET studies in human subjects. J Cereb Blood Flow Metab. 1990 Sep;10(**5**):740–7.

8. Ichise M, Toyama H, Innis RB, Carson RE. Strategies to improve neuroreceptor parameter estimation by linear regression analysis. J Cereb Blood Flow Metab. 2002 Oct;22(**10**):1271–81.

9. Grace AA. Dysregulation of the dopamine system in the pathophysiology of schizophrenia and depression. Nat Rev Neurosci. 2016 Aug;17(**8**):524–32.

10. Pilowsky LS, Bressan RA, Stone JM, Erlandsson K, Mulligan RS, Krystal JH, et al. First in vivo evidence of an NMDA receptor deficit in medication-free schizophrenic patients. Mol Psychiatry. 2006 Feb;11(**2**):118–9.

11. Rubio MD, Drummond JB, Meador-Woodruff JH. Glutamate receptor abnormalities in schizophrenia: implications for innovative treatments. Biomol Ther (Seoul). 2012 Jan;20(**1**):1–18.

12. The FIL Methods Group. Statistical Parametric Mapping software. Wellcome Trust Centre for Neuroimaging. Institute of Neurology, University College London; 2014.
